# Intraoperative OCT-Guided Selective Epiretinal Membrane (ERM) Peeling Versus ERM and Internal Limiting Membrane Peeling for Tractional Macular Edema in Diabetic Eyes

**DOI:** 10.3390/diagnostics14232610

**Published:** 2024-11-21

**Authors:** Francesco Pignatelli, Alfredo Niro, Pasquale Viggiano, Giacomo Boscia, Giuseppe Addabbo, Francesco Boscia, Cristiana Iaculli, Ermete Giancipoli

**Affiliations:** 1 Eye Clinic, Hospital “SS. Annunziata”, ASL Taranto, 74100 Taranto, Italy; pignatelli.oculista@gmail.com (F.P.); pinoaddabbo@tiscali.it (G.A.); 2 Department of Translational Biomedicine Neuroscience, University of Bari “Aldo Moro”, 70125 Bari, Italy; pasquale.viggiano90@gmail.com (P.V.); bosciagiacomo@gmail.com (G.B.); francescoboscia@hotmail.com (F.B.); 3 Department of Ophthalmology, Policlinico Riuniti Foggia, University of Foggia, 71122 Foggia, Italy; cristiana.iaculli@unifg.it (C.I.); ermete.giancipoli@gmail.com (E.G.)

**Keywords:** diabetes, macular edema, intraoperative OCT, epiretinal membrane, macular surgery

## Abstract

Background and Aim: Despite the abundant literature, internal limiting membrane (ILM) peeling remains a controversial topic, especially in diabetic eyes. We compared the safety and effectiveness of intraoperative optical coherence tomography (iOCT)-assisted selective epiretinal membrane (ERM) peeling with dye-assisted ERM and ILM peeling, for the treatment of tractional diabetic macular edema (tDME). Material and Methods: In this single-center retrospective study, we evaluated consecutive patients with tDME who underwent iOCT-assisted selective ERM peeling (Group A) or “dual blue” dye-assisted ERM and ILM peeling (Group B). Best corrected visual acuity (BCVA) and central macular thickness (CMT) were compared over a 12-month follow-up. A linear mixed model analysis was performed. Results: At baseline, the two groups were comparable in terms of their demographic and clinical outcomes. No significant difference between BCVA and CMT was observed among the groups. Both groups showed significant improvement in outcomes at the last follow-up (*p* < 0.001), although only iOCT-assisted ERM peeling ensured significant visual gain and macular thinning (*p* < 0.001) one month after surgery. A significant effect of time on both outcomes (*p* < 0.001) and of time–treatment interaction on visual change (*p* = 0.02) were observed. In eight patients, macular edema recurred (Group A: two patients; Group B: six patients) and was managed with an intravitreal dexamethasone implant. In Group A, one patient developed a recurrence of ERM without the need for reoperation. Conclusions: iOCT-assisted ERM removal may be as effective as dye-assisted ERM and ILM peeling to treat tDME. Additionally, it ensures a quicker recovery of visual function and macular thickness. The observed ERM recurrence within the 1-year follow-up was mild and did not necessitate additional surgery.

## 1. Introduction

Diabetic macular edema (DME) is a well-known and potentially sight-threatening condition, affecting approximately 6.8% of the general population [1] and 20% of the diabetic population [2,3,4].

An epiretinal membrane (ERM), also referred to as a macular pucker, is a fibrous layer that develops on the surface of the retina, specifically in the macular area; it can be idiopathic or secondary to ocular diseases, trauma, or previous intraocular operation [5,6,7].

ERM formation in diabetic eyes with DME has been widely described [8,9], with a varying incidence rate that has been reported as being between 13 and 46% [10,11,12].

It has been suggested that the higher ERM incidence in the diabetic population may be due to advanced glycation and accumulation products that lead to a higher incidence of incomplete posterior vitreous detachment (PVD) and vitreoschisis [13,14].

Pars plana vitrectomy (PPV) and ERM removal are the usual surgical treatments for patients with symptoms [15], and most patients experience better vision after the procedure. However, ERM recurs in approximately 10% to 21% of patients, and 3% of those with recurrence require another surgery [16,17,18].

The efficacy of inner limiting membrane (ILM) peeling, compared to ERM removal, in improving visual function for patients affected by tDME is still unclear, with conflicting results found [19,20].

ILM removal following the removal of the tractional membrane, should restore elasticity to the retina and improve visual function [20].

Furthermore, ILM peeling would lead to less ERM recurrence in eyes with idiopathic ERM [15] and proliferative membrane in diabetic retinopathy [19,20].

Conversely, ILM peeling could harm Müller cells, leading to worse vision, macular edema, or retinal bleeding [21].

Different dyes have been used to stain the ERM, ILM, or both, making peeling safer [22] and the more common treatment option for ERM surgery [23].

Advancements in real-time imaging with iOCT offer detailed information during ERM surgery that is not available when using traditional methods, even when using dyes. Some studies suggested that the complete removal of the idiopathic ERM without dyes is achievable with iOCT and that it leads to improved vision with low ERM recurrence [24,25,26].

However, comparative studies between iOCT and conventional surgery are lacking and focus only on idiopathic ERM [27].

The aim of this study was to compare clinical outcomes between iOCT-guided ERM removal and dye-assisted ERM and ILM removal in diabetic patients with tDME.

## 2. Materials and Methods

We conducted a retrospective, comparative, single-center cohort study on consecutive patients affected by tDME, who were treated with iOCT-assisted selective ERM peeling or dye-assisted ERM and ILM peeling, between January 2022 and July 2023, at the Eye Clinic of “SS. Annunziata” Hospital in Taranto, Italy.

The same expert surgeon (F.P.) performed all surgeries, and iOCT was used when the main operating room was available.

Inclusion criteria encompassed eyes with tDME, defined as central-involved diabetic macular edema (cystoid, sponge-like, or retinal detachment pattern) associated with ERM. Additionally, patients had either non-proliferative diabetic retinopathy or proliferative diabetic retinopathy, previously treated with laser photocoagulation or anti-vascular endothelial growth factor (VEGF) injections. Exclusion criteria included glycated hemoglobin A1c (HbA1c) > 9% (75 mmol/mol), untreated proliferative diabetic retinopathy, a history of ocular hypertension or glaucoma, previous or concomitant retinal diseases (including retinal vein occlusion and age-related macular degeneration), recent treatment of DME with intravitreal anti-VEGF or corticosteroids, recent cataract surgery, prior vitreoretinal surgery, and incomplete follow-up.

The primary outcomes were best-corrected visual acuity (BCVA) and central macular thickness (CMT) over a 12-month follow-up. The recurrence rate was also assessed as a secondary outcome.

This retrospective study adhered to the ethical standards of the institutional and national research committee, as well as the 1964 Helsinki Declaration and its later amendments. The study was approved by the Institutional Review Board (IRB) of the Eye Clinic of “SS. Annunziata” Hospital in Taranto, Italy.

### 2.1. Surgical Procedure

All patients underwent 25-gauge three-port vitrectomy using the Constellation “Vision System” (Alcon Laboratories, Fort Worth, TX, USA). Preoperative preparation included povidone iodine 5% application and peribulbar anesthesia. Conjunctival displacement and three oblique incisions facilitated the insertion of valved cannula trocar systems. Central and peripheral vitrectomy was performed at 7500 cuts per minute with linear aspiration. For posterior visualization, in Group A, an operative microscope with integrated OCT (Proveo 8, Leica, Welzar, Germany) with Oculus BIOM 5 (Oculus Surgical Inc., Port St. Lucie, FL, USA) and a plano-concave contact lens was used, while, in Group B, the Opmi Lumera 700 (Carl Zeiss, Jena, Germany) with Resight 700 (Carl Zeiss, Jena, Germany) and a plano-concave contact lens was used.

In Group A, ERM peeling was started at the edge of the epiretinal membrane according to preoperative OCT scans, which were then confirmed using intraoperative OCT Live Scan. We avoided using staining solution, but multiple intraoperative scans were carried out during the whole procedure. A whole posterior pole scan was then performed at the end of the peeling to make sure no cortical or ERM remnants were left.

In Group B, ERM and ILM peeling were performed in all patients after staining with Trypan Blu (TB) 0.15%  +  Brilliant Blue G (BBG) 0.05%  +  Lutein 2% solution (DOUBLE DYNE; Alfa Intes Industria Terapeutica Splendore S.r.l., Naples, Italy). The initial grasping site was determined during surgery based on the intraoperative visualization of the membrane’s edge after staining.

For both groups, 25-G internal limiting membrane forceps (Alcon Laboratories, Fort Worth, TX, USA) were used. A sclerotomy site suture was performed only when needed due to leakage of the wound.

### 2.2. Assessments

All participants underwent a comprehensive ophthalmic examination at baseline, which included the collection of demographic and medical history data, as well as HbA1c levels. Best-corrected visual acuity (BCVA), intraocular pressure (IOP), and central macular thickness (CMT) were measured at the time of surgery (T0) and during follow-up visits at 1, 3, 6, and 12 months. IOP was measured using a Goldmann tonometer, and BCVA was assessed using a standardized Early Treatment Diabetic Retinopathy Study (ETDRS) protocol, with ETDRS values converted to the logarithm of the minimum angle of resolution (logMAR) for statistical analysis. CRT was evaluated using spectral-domain optical coherence tomography (SD-OCT; CIRRUS, Carl Zeiss, Jena, Germany) and defined as the average thickness of the macula within the central 1 mm ETDRS grid. Central involved macular edema was defined as a CMT greater than 300 microns. IOP measurement and all intraoperative and postoperative adverse events were recorded for safety evaluation.

### 2.3. Statistical Analysis

For the description of patients’ characteristics at baseline, mean ± standard deviation (SD) was used for continuous variables and counts with percentages for categorical variables. Demographic and baseline characteristics of the two samples were compared using Fisher’s exact test for categorical variables and the Mann–Whitney U Test for quantitative ones.

A linear mixed model was used to evaluate repeated measurements of BCVA and CMT at each time point within each group and among the groups, and the trajectories of BCVA and CMT.

Mean IOP, as the safety parameter, was evaluated within and among the groups over follow-up. A *p*-value < 0.05 was considered statistically significant. No formal sample size calculation was performed. All statistical analyses were performed using the software package SAS (SAS Analytics Software, Cary, NC, USA) (version 9.1 or higher).

## 3. Results

The study enrolled 36 patients; 17 were assigned to Group A and 19 to Group B. There were nine female patients in both groups. The overall mean age was 71.1 ± 6.2 years (range, 85–59 years); more than 90% of patients had type 2 diabetes. No significant difference in HbA1c was observed between the groups. The overall mean time of diabetes was 16.5 ± 4.8 years (range, 5–26 years). All patients were pseudophakic. No significant difference in demography was observed between the groups. There were no significant differences between the two groups regarding mean BCVA, CMT, and IOP at baseline (Table 1).

The mean BCVA, CMT, and IOP values at each time point [1 month (T1), 3 months (T2), 6 months (T3), and 12 months (T4)] were compared to the baseline (T0) value within each group. None of the patients missed follow-up visits.

**Figure 1:**
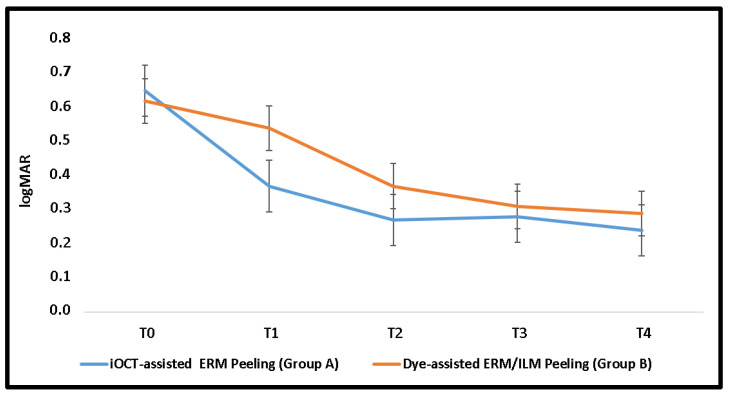
Best corrected visual acuity of both groups over follow-up.

**Figure 2:**
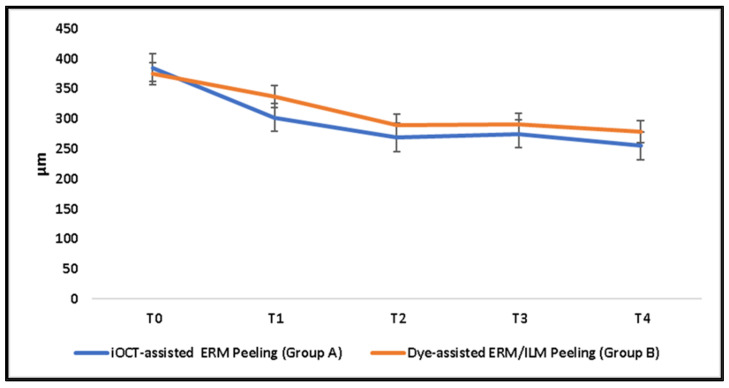
Central macular thickness of both groups over follow-up.

Both groups showed significant improvement in BCVA and CMT (*p* ≤0.001) as early as the 3-month follow-up. In Group A, a significant visual gain and macular thinning (*p* ≤ 0.001) occurred just one month after surgery. No significant difference between BCVA and CMT was observed among the groups over follow-up. (Table 2; Figure 1 and Figure 2)

The overall mean visual gain at the last follow-up was 0.34 ± 0.21 logMAR (range, −0.22 logMAR to 0.78 logMAR). The visual gain in Group A (0.35 ± 0.20 logMAR) was not significantly different from the visual recovery in Group B (0.34 ± 0.25 logMAR) (*p* = 0.81). In each group, only one patient had worse BCVA at the last follow-up than at the baseline.

The overall mean thickness reduction at the last follow-up was 108.7 ± 87.2 µm (range, −85 µm to 311 µm). The mean reduction in CMT in Group A (132.8 ± 80.01 µm) was greater than that in Group B (87.1 ± 89.7), although not significantly. In Group B, two patients observed an increase in CMT at the last follow-up, compared to the baseline value. In eight patients (Group A, two patients; Group B, six patients), macular edema recurred over the follow-up period and was managed with an intravitreal dexamethasone implant. In Group A, one patient developed a recurrence of ERM without the need for reoperation.

**Figure 3:**
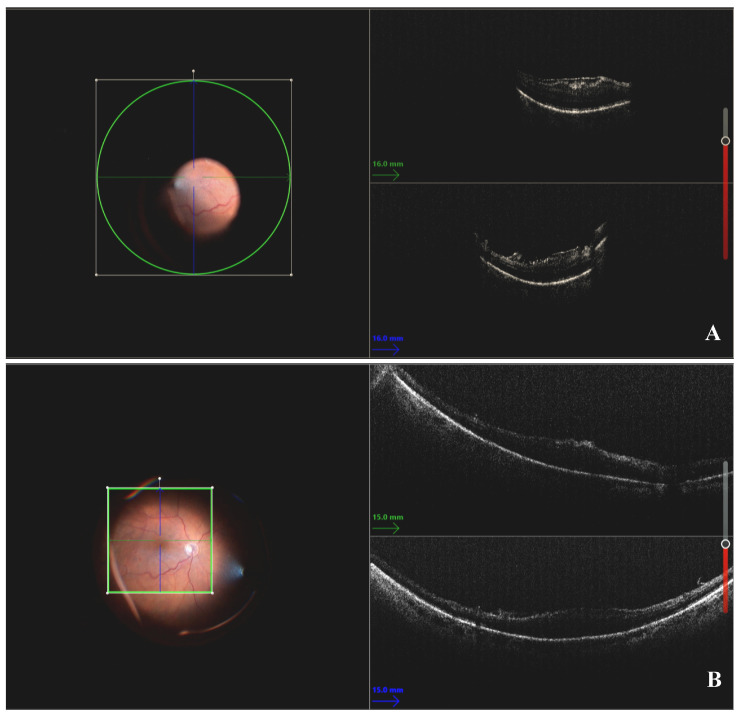
(**A**) Intraoperative fundoscopic image of macular site before epiretinal membrane (ERM) peeling (left side); single OCT scans on horizontal (green line) and vertical (blue line) axes show ERM as hyperreflective band on retinal surface (right side). (**B**) Intraoperative fundoscopic image of macular site after ERM peeling (left side); OCT scans highlighted macular site after ERM removal without need for additional staining.

A representative case highlighting selective ERM peeling using iOCT is shown in Figure 3.

In the linear mixed model analysis, a significant effect of time on both outcomes (*p* ≤ 0.001) and of time–treatment interaction on visual change (*p* = 0.02) were observed (Table 2).

### Intraocular Pressure and Complications

During the study, the mean IOP did not significantly increase in either group; neither were significant differences observed between the groups (Table 3). None of the patients showed a significant increase in their IOP requiring medical or surgical management. No surgical complications were detected.

## 4. Discussion

In the treatment of tDME, both surgical approaches, including iOCT-assisted selective ERM peeling and dye-assisted ERM and ILM peeling, ensure a remarkable visual recovery and macular thickness reduction after a 12-month follow-up, with no difference in the amount of morpho-functional improvement between the two. In the linear mixed model, we assessed the impact of each treatment, time, and their interaction on outcomes. No significant differences among the groups were observed for repeated measurements of BCVA and CMT over the course of one year. The group that underwent iOCT-guided peeling experienced a significant improvement in visual function and macular thickness at all time points, while those who underwent dye-assisted peeling saw improvements only three months after surgery. We noted a significant effect of time on both outcomes and of treatment–time interaction on visual function specifically.

In line with previous results that have shown good functional and morphologic recovery after the removal of ERM with or without ILM peeling for the surgical management of ERM in diabetic or non-diabetic retinopathy [19,20,21], we observed that the final recovery of ERM combined with or without ILM peeling was similar, although the iOCT-guided ERM removal without ILM peeling ensured an early better morpho-functional recovery compared to the presurgical condition.

Although short-and long-term visual function improved similarly in the patients who underwent or did not undergo ILM peeling, the short-term macular thickness had a more remarkable decrease when ILM peeling was not performed, and visual recovery was mainly linked to ERM removal [21]. Furthermore, clinically significant macular edema was more likely to recur in patients who underwent ILM peeling (31.6%) compared to those in the iOCT–guided ERM removal group (11.8%). Huang et al. [21] and Chang et al. [28] reported in two different meta-analyses that the reduction in central thickness during a 12-month follow-up was more relevant in the group without ILM peeling. Also, although an additional ILM peeling did not significantly affect morpho-functional outcomes recovery compared with the absence of ILM peeling, as reported by Azuma et al. [29], who analyzed 15 studies on idiopathic epiretinal membrane, the efficacy of ILM peeling in tDME management tends to decrease over time. Consequently, a small but variable percentage of patients may require additional treatments, including intravitreal dexamethasone implants, as previously reported [30,31,32,33,34].

Although the ILM is pathologically thickened in diabetic eyes due to the accumulation of extracellular matrix elements by macrophages and fibroblasts and although its removal allows the release of tangential tractional forces [35] and the removal of advanced glycation end-product receptors whose activation stimulates VEGF upregulation and thereby exacerbates DME [36], ILM peeling may also have potential side effects due to mechanical injury to the retina. These side effects can include the disorganization of the inner retinal layers with the formation of cystic spaces, which may result from late-onset Müller cell death [37], and photoreceptors defects [38]. The disorganization of the inner retinal layers could lead to reactive macular edema that may develop from the aforementioned cystic spaces [37], causing microscotomas and a slow recovery of retinal sensitivity [39,40], while outer retina defects cause slower early visual acuity recovery [38].

Dye-assisted macular peeling became the state-of-the-art technique for peeling ILM, protecting the retina from mechanical injury during the procedure and increasing the surgeons’ confidence in performing the surgery [21,41]. In line with the standard of care, we preferred using a dye solution with TB, BBG, and lutein to provide very effective staining of ERM and ILM and a protective effect for the macular structure [42].

In light of the recent literature, which has reported on the safety and effectiveness of iOCT-guided ERM peeling [25,26] and on similar outcomes of iOCT-guided surgery without ILM peeling when compared with conventional surgery [27,43], we first compared a standard surgical approach, including ILM peeling, with iOCT-guided ERM peeling in eyes affected by tDME.

As mentioned above, both treatments improved the morpho-functional status at the macular site, but iOCT-guided peeling leads to earlier functional recovery, particularly in the first few months after surgery. iOCT intraoperatively allows for the in vivo assessment of the retinal architecture and tissue planes and for the manipulation of the macular site with targeted maneuvers [44]. In the dye-assisted peeling group, the site with better visualization of ERM via staining was chosen as the initial grasping site; therefore, the grasping sites were potentially located in different places. Conversely, in the iOCT-guided peeling group, the grasping site was identified at the edge of the membrane based on a double check between preoperative and intraoperative OCT scans, allowing a more objective approach to the macular site. Furthermore, in the iOCT-guided peeling group, ILM staining after ERM peeling was generally not performed, since complete ERM removal, the goal of surgery, was carefully confirmed using iOCT, avoiding the use of dye. Minimizing the use of dye during surgery could enhance surgical efficiency and reduce the need for extensive manipulation, which carries small but inherent risks, such as transretinal dye penetration. Furthermore, iOCT allowed for additional membrane peeling in 12% of cases and helped avoid unnecessary surgical maneuvers in 9.2% of cases [25].

The targeted and precise maneuvers at the macular site, guided by iOCT, potentially reduce surgical trauma on retinal tissue. This may lead to a quicker recovery of macular thickness and a lower rate of edema recurrence, positively affecting visual recovery.

If iOCT-guided ERM removal provided similar anatomic and functional results to conventional ERM, the support of iOCT during surgery was described as really helpful in identifying the edge of the membrane to start peeling, confirming the complete peeling or performing additional peeling and avoiding additional staining [24,25,26,27,43].

Consistent with previous findings on idiopathic ERM peeling [45,46,47], the rate of ERM recurrence after surgery was very low. Only one patient who underwent iOCT-assisted ERM peeling showed membrane recurrence during the follow-up period but did not need a second surgery. Our results confirmed that ERM recurrence not requiring surgery occurred more frequently after iOCT-guided peeling, as previously reported [27], probably due to the limitation of iOCT in visualizing small ERM components or a subtype of ERM that originates with the ILM and contains no contractile elements [48,49].

Although we know that the removal of the ILM decreases the risk of recurrence of ERM due to its potential role as a scaffold for epiretinal proliferation [50,51], our results might confirm that the ILM peeling is not all that necessary in preventing ERM recurrence requiring a surgical approach, as previously suggested [27].

To the best of our knowledge, this is the first study to select only diabetic eyes with ERM and macular edema.

Another crucial aspect is the duration of diabetes, a high HbA1c level at the time of surgery, and visual acuity at baseline, which are known to be associated with poor functional recovery after surgery [52,53]. So, our functional results should be analyzed while considering that potentially worse cases (i.e., HbA1c > 9%) were excluded, and that the mean duration of diabetes mellitus and the mean visual acuity before surgery were not significantly different between the groups.

This study has some additional limitations that should be noted. One is its retrospective design. Other limitations are the single-center nature of the study, the limited number of patients, and the absence of analysis of retinal layer integrity and its relationship with visual function. Additionally, iOCT cannot determine if the peeling was solely due to ERM peeling or a combination of ERM and ILM peeling in the iOCT group, leading to inherent heterogeneity within this group.

In conclusion, iOCT-guided ERM removal and dye-assisted ERM and ILM peeling showed remarkable safety and effectiveness in treating tDME over a long follow-up. Visual function and macular thickness improved regardless of the treatment, although an early recovery was reported after iOCT-guided peeling. Our comparative results suggest an alternative approach to tDME when diagnostic tools such as iOCT are available.

## Figures and Tables

**Table 1:** Demographic and baseline clinical characteristics of study population.

	Group A (*n* = 17)	Group B (*n* = 19)	*p*
Age, years	69.8 ± 5.6	72.3 ± 6.6	0.2
Sex			
Male	8 (47.1)	10 (52.6)	
Female	9 (52.9)	9 (47.4)	0.9 *
Type 2 DM	15 (88.2)	18 (94.7)	
Type 1 DM	2 (11.8)	1 (5.3)	0.9 *
Glycated hemoglobin (%)	6.8 ± 0.8	7.04 ± 0.7	0.2
Years from diagnosis of diabetes	16.2 ± 3.6	16.7 ± 5.8	0.9
BCVA, logMAR	0.65 ± 0.19	0.63 ± 0.24	0.7
CMT, microns	385 ± 81.4	375 ± 68	0.8
IOP, mmHg	15.8 ± 1.3	15.8 ± 1.5	0.8

Abbreviations: DM, diabetes mellitus; BCVA, best corrected visual acuity; CMT, central macular thickness; IOP, intraocular pressure. Unless otherwise indicated, values are mean ± SD or no. (%). *p*, Mann-Whiney U test; *, Fisher’s exact test.

**Table 2:** Linear mixed model analysis to examine the effect of different treatments on BCVA and CMT in different time points (*n* = 36).

Parameters *	Time					*p* ^¥^			
	T_0_ (a)	T_1_ (b)	T_2_ (c)	T_3_ (d)	T_4_ (e)	b vs. (a)	c vs. (a)	d vs. (a)	e vs. (a)
BCVA									
Group A	0.65 ± 0.19	0.37 ± 0.17	0.27 ± 0.15	0.28 ± 0.20	0.24 ± 0.13	<0.001	<0.001	<0.001	<0.001
Group B	0.63 ± 0.24	0.54 ± 0.32	0.37 ± 0.20	0.31 ± 0.22	0.29 ± 0.16	0.99	<0.001	<0.001	<0.001
*p* ^^^	0.69	0.06	0.15	0.57	0.35				
Mixed ^§^	Treatment 0.28	Time <0.001	Interaction 0.02						
CMT									
Group A	385 ± 81.4	302 ± 43.6	269 ± 35.7	275 ± 63.7	255 ± 22.9	<0.001	<0.001	<0.001	<0.001
Group B	375 ± 68.0	337 ± 77.0	289 ± 69.2	291 ± 81.0	279 ± 57.3	0.33	<0.001	<0.001	<0.001
*p* ^^^	0.72	0.11	0.37	0.58	0.32				
Mixed ^§^	Treatment 0.31	Time <0.001	Interaction 0.29						

* As mean and standard deviation (mean ± SD). T_0_, baseline; T_1_, month 1; T_2_, month 3; T_3_, month 6; T_4_, month 12. Abbreviation: BCVA, best corrected visual acuity; CMT, central macular thickness. ^^^: treatment effect for each time; ^§^: mixed-effects; ^¥^: contrasts of marginal linear predictions.

**Table 3:** Mean intraocular pressure in both groups over follow-up.

	Time					*p* ˆ			
	T_0_ (a)	T_1_ (b)	T_2_ (c)	T_3_ (d)	T_4_ (e)	b vs. (a)	c vs. (a)	d vs. (a)	e vs. (a)
Group A *	15.8 ± 1.35	15.8 ± 1.6	15.4 ± 1.6	17.0 ± 3.6	16.1 ± 1.7	0.99	0.99	0.99	0.99
Group B *	15.8 ± 1.47	16.0 ± 1.1	16.2 ± 0.8	16.7 ± 1.2	17.3 ± 1.7	0.99	0.99	0.99	0.20
*p* ^†^	0.82	0.61	0.06	0.49	0.05				

* As mean and Standard Deviation (mean ± SD). T_0_, baseline; T_1_, month 1; T_2_, month 3; T_3_, month 6; T_4_, month 12. Mann–Whitney test: *p* ˆ, within each group, *p* ^†^, among the groups.

## Data Availability

All raw data are available on request.
